# Bryant S. Traywick

**Published:** 1879-06

**Authors:** 


*Bryant S. Traywick, D. D. S.*, of Monroe, North Carolina, and a Graduate of the Baltimore College of Dental Surgery, of the class of 1860, was, a few weeks ago, found dead in his office. The cause of his death was supposed to be heart disease, symptoms of which manifested themselves several years ago.

Dr. Traywick was a respected member of the community in which he lived, and enjoyed during his twenty years of professional life a lucrative practice.

